# Grip Strength, Anthropometric Indices, and Their Combination in Screening for Metabolic Syndrome in the Korean Population

**DOI:** 10.3390/jcm13195988

**Published:** 2024-10-08

**Authors:** Bum Ju Lee

**Affiliations:** Digital Health Research Division, Korea Institute of Oriental Medicine, Daejeon 34054, Republic of Korea; bjlee@kiom.re.kr; Tel.: +82-42-868-9593

**Keywords:** metabolic syndrome, hand grip strength, risk factor, anthropometry, association

## Abstract

**Background**: Metabolic syndrome (MS) is a cluster of cardiometabolic risk factors for future diabetes and cardiovascular diseases, and low grip strength (GS) is associated with an increased risk of MS. However, the index (among absolute GS, relative GS, and anthropometric indices) that is more strongly associated with MS has not been conclusively identified. Therefore, the objective of the present study was to simultaneously examine the associations of MS with relative and absolute GS and anthropometric indices in a Korean population. **Methods**: In this large-scale cross-sectional study, we used data from the Korean National Health and Nutrition Examination Survey (KNHANES). A total of 20,915 subjects were included in the statistical analyses. Complex sample binary logistic regression models were used to analyze the associations between MS and indices such as the waist-to-height ratio (WHtR), body mass index (BMI), and absolute and relative GS. **Results**: The prevalence of MS was 40.48% in men and 34.4% in women. The mean GS values in the healthy group and MS group were 38.06 ± 0.13 and 38.06 ± 0.15 years for men (*p* = 0.980) and 22.72 ± 0.07 and 21.19 ± 0.11 years for women (*p* < 0.001), respectively. Among all the indices, the WHtR was the index most strongly associated with MS in men. Among the GS indices, the GS/weight index was closely associated with MS, and the magnitude of this association was stronger than that of the absolute GS index. In women, the WHtR was the most related index to MS among all the indices. Among the GS indices, the GS/weight and GS/BMI indices were strongly associated with MS, and the magnitudes of these associations were much greater than those of the absolute GS index. **Conclusions**: Although absolute and relative GS indices are strongly associated with MS in the Korean population, the strongest association was observed for the simple anthropometric index (WHtR).

## 1. Introduction

Metabolic syndrome (MS) is a critical predictor or trigger of type 2 diabetes and cardiovascular diseases [1,2,3,4]. The prevalence of MS increased from 36.2% to 47.3% in U.S. adults from 1999 to 2019 [2]. MS consists of five components, including elevated blood pressure, abdominal or central obesity, low high-density lipoprotein (HDL) cholesterol levels, hypertriglyceridemia, and elevated glucose levels, and is diagnosed when three or more of the components are present [1,2,3,4]. In terms of clinical significance, the ultimate importance of MS is to reveal individuals with a high risk of cardiovascular diseases and diabetes [4] because, compared with individuals without MS, individuals with MS have a 2-fold greater risk of developing cardiovascular diseases within the next 10 years [1]. Risk factors are associated with MS, including dietary habits, physical activity status, sleep duration, obesity status, smoking status, depression status, osteoarthritis status, education and income levels, genetics, ethnicity, and muscle strength and mass [2,3].

In general, anthropometric indices are used to screen for obesity and obesity-related diseases, and grip strength (GS) measurements are used to screen for muscle strength/function and muscle-strength-related diseases. Recently, many studies reported that diseases such as MS, hypertension, diabetes, anemia, myocardial infarction, and arthritis are closely associated with GS and anthropometric indices [5,6,7,8,9,10,11,12,13,14,15,16,17,18,19,20], and GS is an indicator used to screen for MS [5,6,7,8,9,10,11,12,13,14,15,16,17]. The widely known anthropometric indices are body mass index (BMI), waist circumference (WC), and the waist-to-height ratio (WHtR). BMI is computed by dividing weight by height squared, and the WHtR is calculated by dividing the WC by the height. In many studies, the WHtR has been considered a predictor of cardiovascular diseases, anemia, and arthritis [18,19,20]. Additionally, previous studies reported that low GS or muscle strength was significantly associated with MS and MS components [5,17]. Furthermore, recent studies have suggested that MS and cardiometabolic disorders are more strongly associated with relative GS indices, such as the GS/BMI [6,10], GS/weight [5,7,9], and GS/body fat mass [8] indices, than with absolute GS indices or anthropometric indices.

Although many studies have suggested that GS is associated with MS, the index (among absolute GS, relative GS, and anthropometric indices) that is more strongly associated with MS has not been conclusively identified. Therefore, the objective of the present study was to simultaneously examine the associations of MS with relative GS, absolute GS, and anthropometric indices in the Korean population.

## 2. Materials and Methods

### 2.1. Study Population

For this large-scale cross-sectional study, we used data from the Korean National Health and Nutrition Examination Survey (KNHANES) from 2014 to 2019, which was conducted by the Korea Disease Control and Prevention Agency (KDCA). The KNHANES is a nationally representative survey of the South Korean population and provides reliable statistics of socioeconomic and demographic characteristics, health-related behavior and dietary intake, biochemical and clinical profiles, and physical examination data [21,22,23,24]. The KNHANES was performed with the approval of the Institutional Review Board of the KDCA (IRB: 2013-12EXP-03-5C, 2018-01-03-P-A, 2018-01-03-C-A) [22,24], and the Institutional Review Board of the Korea Institute of Oriental Medicine approved the use of KNHANES data (IRB No. I-2209/009-001). All participants in the survey provided written informed consent. The present study was conducted in accordance with the principles of the Helsinki Declaration, and all materials and statistical methods were selected and performed in accordance with the guidelines of the KDCA [22,23,24].

**Figure 1:**
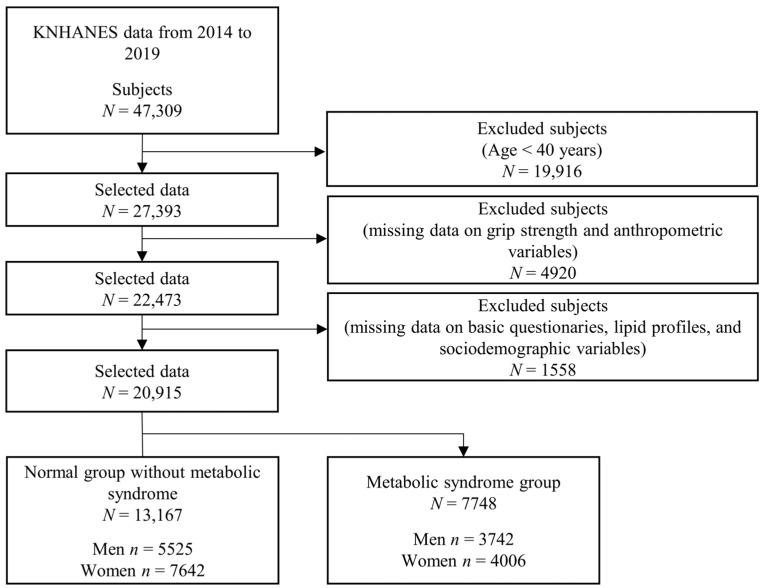
Sample selection process.

A total of 47,309 subjects participated in the KNHANES from 2014 to 2019. With respect to inclusion and exclusion, we focused on middle-aged and older subjects (<40 years) because the population included very few subjects with MS aged under 40 years. We excluded subjects with missing data on GS and anthropometric variables, lipid profiles, sociodemographic variables, important questionnaire responses, and variables related to MS. Overall, 20,915 subjects were included in the statistical analyses (9267 men and 11,648 women). More specific information on the inclusion and exclusion criteria and the number of subjects are presented in Figure 1.

### 2.2. Definition of Metabolic Syndrome

MS was defined according to the National Cholesterol Education Program Adult Treatment Panel III (NCEP ATP III) and the World Health Organization (WHO) recommendations [25,26]. Subjects with at least three of the following five components were considered to have MS: (1) a triglyceride (TG) level of ≥150 mg/dL (1.69 mmol/L) or the use of triglyceride-lowering medications, (2) a high-density lipoprotein (HDL) cholesterol level of <40 mg/dL (1.04 mmol/L) in men and <50 mg/dL (1.29 mmol/L) in women or the use of cholesterol-lowering medication, (3) systolic and diastolic blood pressures (SBPs and DBPs) of ≥130/85 mmHg or the use of blood pressure medication, (4) a fasting plasma glucose (FPG) of ≥110 mg/dL (≥6.1 mmol/L) or the use of glucose-lowering medication, and (5) a WC of >90 cm in men and >80 cm in women. We used WC values >90 cm for men and >80 cm for women because the WHO and previous studies recommended these ethnicity-specific values for South Asian and Chinese individuals [4,26].

### 2.3. Measurements and Laboratory Tests

GS and anthropometric indices were measured by experts and well-trained staff via standardized measurement protocols. GS (kg) was obtained via a digital grip strength dynamometer (T.K.K 5401, Japan; Takei Scientific Instruments Co., Ltd., Tokyo, Japan). Subjects with a history of hand or arm surgery during the last 3 months and/or pain at the measurement points within the last 7 days were excluded from the GS measurements. During the measurement, all the subjects stood with their feet shoulder-width apart and with their elbows and wrists not touching the body, and the GS measurement was repeated three times. The absolute GS index was obtained by the maximum value in the dominant hand. For comparisons with the relative GS indices reported in previous studies, we calculated relative GS indices by dividing GS by weight (kg), BMI (kg/m^2^), and the WHtR (waist-to-height ratio) (namely, the GS/weight, GS/BMI, and GS/WHtR indices, respectively). The detailed configuration of the GS measurements is described in the literature [18,19,20].

Anthropometric data were obtained by well-trained medical experts via standardized protocols. Weight and height (cm) data were obtained via automatic measurement equipment (JENIX DS-102, Dong Sahn Jenix Co., Seoul, Korea). WC was obtained to the nearest 0.1 cm using flexible plastic tape (Seca 200, Hamburg, Germany) at the midline between the lower rib margin and the iliac crest. BMI was obtained by dividing weight (kg) by height squared (m^2^), and the WHtR was calculated by dividing WC by height.

For biochemical profiles, blood samples were obtained after fasting for at least 8 h. FPG (mg/dL), HDL cholesterol (mg/dL), and TG (mg/dL) levels were analyzed via a Hitachi Automatic Analyzer 7600-210 (Hitachi, Tokyo, Japan) and a Labospect008AS (Hitachi/Japan). SBP and DBP were measured three times via a standard mercury sphygmomanometer (Baumanometer Wall Unit 33-0850, WA Baum Co., Copiague, NY, USA), and the average value of the second and third measurements was used.

### 2.4. Covariates

The sociodemographic and socioeconomic characteristics of all the participants were collected and are described in Table 1. We selected the relevant covariates on the basis of previous studies [5,6,7,8,9,10,11,12,13,14,15,16] that focused on GS and MS/metabolic abnormalities. The relevant covariates were as follows: age [5,6,7,8,9,10,15,16], geographic area [9,10], education level [5,8,10,16], occupation type [8,15], household income level [5,8,10,16], stress status [11,12], alcohol intake [7,8,9,10,15,16], smoking status [7,8,9,10,15,16], and physical activity status [6,8,10,16]. Additionally, menopause was used as a covariate only for women [13,14]. Detailed information on all covariates is described in Table 1.

### 2.5. Statistical Analysis

The KNHANES was designed with complex survey samples to represent the entire Korean population, and data clustering, weighting, and stratification were applied. Therefore, statistical analyses were performed via complex sample analysis procedures. The detailed complex survey sample design was described in previous studies [18,19,20,22]. Sex differences were calculated via Rao–Scott chi-square tests for categorical variables and *t* tests with general linear models for continuous variables. To assess the associations of MS with GS and anthropometric indices, we used complex sample binary logistic regression. We built three models based on the relevant covariates: an unadjusted model (crude model), a model adjusted for age (Model 1), and a model adjusted for age, geographic area, education level, occupation type, household income level, stress status, alcohol intake, smoking status, physical activity status, and menopause status (only in women) (Model 2). Prior to the statistical analysis, we checked the variance inflation factor to avoid potential multicollinearity between indices or variables and tested the linearity between the logit of independent and dependent variables based on the Box–Tidwell test. All the statistical analyses were conducted with IBM SPSS Statistics 28 (IBM SPSS, Inc., Chicago, IL, USA). We considered a statistical significance level of 0.05 and odds ratios with 95% confidence intervals (CIs).

## 3. Results

Table 1 presents the basic characteristics of the subjects who participated in this study. In terms of sex differences, all variables, except for dominant hand status, showed substantial and moderate differences according to sex. With respect to the associations between the healthy and MS groups, the following variables were strongly associated with MS (*p* < 0.001): age, anthropometric indices, GS indices, geographic area, education level, occupation type, household income level, smoking status, alcohol intake, stress status, physical activity status, FPG level, HDL cholesterol level, TG level, metabolic components (high BP, FG, TG, and WC measurements and low HDL cholesterol), and menopausal status (only women); however, height (*p* = 0.034), absolute GS (*p* = 0.980), and dominant hand status in men (*p* = 0.573) and women (*p* = 0.572) were not associated with MS.

Table 2 shows the associations of MS with the anthropometric, absolute GS, and relative GS indices in men. Among all the indices, the WHtR was the most strongly associated with MS in all the models (OR = 3.98 [3.65–4.35], *p* < 0.001 in the crude model; adj. OR = 3.99 [3.65–4.36], adj. *p* < 0.001 in Model 1; adj. OR = 4.08 [3.72–4.46], adj. *p* < 0.001 in Model 2). The absolute GS index was not associated with MS in the crude model, but, in Models 1 and 2, the GS was associated with MS. Among the relative GS indices, the GS/weight index was closely associated with MS, and the magnitude of the association was stronger than that of the absolute GS index.

Table 3 shows the associations of MS with anthropometric, absolute GS, and relative GS indices in women. Among all indices, the WHtR was the most related to MS (OR = 5.27 [4.87–5.71], *p* < 0.001 in the crude model; adj. OR = 4.49 [4.14–4.87], adj. *p* < 0.001 in Model 1; adj. OR = 4.40 [4.05–4.79], adj. *p* < 0.001 in Model 2). The absolute GS index was associated with MS in all the models. Among the relative GS indices, the GS/weight and GS/BMI indices were strongly associated with MS, and the magnitudes of these associations were much greater than that of the absolute GS. Overall, although the absolute GS and relative GS indices were strongly associated with MS in both men and women, the highest associations between GS and all indices were observed for the simple anthropometric index (WHtR) in both men and women.

## 4. Discussion

Among all the anthropometric and GS indices, the WHtR index was the index most strongly associated with MS in the Korean population. Among the GS indices, the GS/weight index was more closely associated with MS than the other relative GS indices and absolute indices in men; in women, the GS/weight and GS/BMI indices were more strongly associated with MS than the other GS indices. Although the absolute and relative GS indices were strongly associated with MS in both men and women, the association was strongest for the simple anthropometric index (WHtR) because the magnitude of the association between the WHtR and MS was much greater than that between all other indices and MS.

### 4.1. Comparison with Previous Studies

Many previous studies have reported that MS is related to muscle strength (GS) [5,6,7,8,9,10,11,12,13,14,15,16,17]. For example, Lopez-Lopez et al. [5] examined the associations of MS with muscle strength and anthropometric indices on the basis of the Prospective Urban Rural Epidemiological (PURE) study and reported that a high WC was an important risk factor for MS and that the relative GS (GS/weight) was a better predictor of metabolic alterations than the GS or the anthropometric index alone. d’Avila et al. [17] investigated the association between low GS and MS in older adults in a systematic review and reported that low GS was significantly correlated with MS and its components in many studies. Wu et al. [16] assessed the association between GS and MS and tested the predictive power of GS for the identification of Chinese adults with MS. They reported that GS was negatively associated with MS, and the receiver operating characteristic (ROC) curve values were 0.65 for men and 0.71 for women. Lee et al. [6] evaluated the relationships of cardiometabolic risk with absolute GS and relative GS (GS/BMI index) in Taiwan and reported that relative GS was more strongly associated with cardiometabolic risk than absolute GS. Kawamoto et al. [7] tested the association between relative GS (GS/weight index) and MS in Japanese community-dwelling people and reported that relative GS was inversely related to an increased risk of cardiometabolic diseases. Song et al. [8] examined the associations of eight relative GS indices with MS and its components in Chinese community-dwelling elderly individuals. They reported that among the GS/weight, GS/BMI, GS/body fat mass, GS/percent body fat, and GS/skeletal muscle mass indices, the best index associated with MS and its components was the GS/body fat mass. Shen et al. [9] investigated the relationship between the relative GS (GS/weight) and the occurrence of MS in China. They argued that relative GS was negatively associated with an increased risk of MS and abdominal obesity and that relative GS was a simple and useful predictor of the development of MS. In South Korea, Yi et al. [10] tested the association between MS and relative GS and reported that relative GS (GS/BMI index) was highly associated with the risk of MS in Korean adults and could be a new biomarker for screening MS. Our findings were consistent with the results of previous studies suggesting that the relative or absolute GS index was strongly associated with MS [5,6,7,10,16,17]. However, our findings disagreed with those of previous studies indicating that the GS/weight and GS/BMI indices were more strongly associated with MS than other relative GS or anthropometric indices were to screen for MS [5,6,7,10]. Our findings showed that the WHtR had the strongest association with MS among all the GS and anthropometric indices in both men and women. With respect to the covariates used in this study, education and income levels were related to a higher risk of MS in women [27,28,29]. Subjects with low education and income levels are more likely to have MS, stroke, and obesity [28]. We assume that education and income levels are important indicators of health and may influence health services, psychosocial stress, and health-related behaviors, such as smoking and drinking [27,29]. Additionally, MS was associated with geographic area. MS is more prevalent in urban areas than in rural areas [30,31]. Therefore, socioeconomic and sociodemographic factors, such as region, sex, income level, and education level, should be considered in studies of MS. In clinical practice, we recommend the use of anthropometric indices rather than GS indices to screen for MS because anthropometry is a better indicator of the disease than GS and is a simple, easy-to-use, and cost-effective measurement.

The exact biological or pathophysiological mechanisms that link GS or muscle strength with MS remain difficult to completely explain [17]. However, partial mechanisms or explanations for the links may be suggested. Sarcopenia due to reduced muscle strength or GS is associated with increased insulin resistance, and increased insulin resistance may lead to MS or diabetes [9,17,32]. Conversely, increased muscle strength developed through physical activity contributes to insulin sensitivity [33]. Additionally, insulin resistance, which occurs in muscle tissue and adipose tissue, plays an important role in the mechanism of abdominal or visceral obesity, as measured by waist circumference, induces an increase in very-low-density lipoproteins and a decrease in HDL, and leads to high blood pressure or hypertension, hypo-HDL cholesterolemia, and cardiovascular diseases [34,35,36]. Furthermore, inflammatory factors are closely linked to MS or low muscle strength [35,37,38,39,40]. The anti-inflammatory cytokine interleukin (IL)-10 is strongly and negatively associated with muscle strength and is positively related to obesity [41], and low IL-10 levels are related to MS [37]. Additionally, changes in IL-10 levels are negatively associated with various metabolic disorders, such as high BP, obesity, high HDL and LDL concentrations, dyslipidemia, and glucose intolerance [39,42]. High levels of the proinflammatory cytokines tumor necrosis factor-α and IL-6 are associated with sarcopenia due to low GS and muscle mass and may exacerbate insulin action [40,42].

### 4.2. Limitations

This study has three limitations. Due to the cross-sectional design of the study, we cannot infer causality from our findings. Moreover, the generalizability of our findings to other ethnic groups or countries cannot be guaranteed because of the differences in ethnicity or countries associated with MS or MS components [1,5]. Because the data used in this study were primarily obtained via questionnaires, the data may be subject to recall bias. To overcome recall bias, the questionnaire was administered in face-to-face interviews conducted by well-trained staff or experts according to established protocols. Despite these limitations, the statistical results of this study are strong and reliable because the KNHANES is a nationally representative survey of a very large Korean population.

## 5. Conclusions

In this study, we demonstrated that absolute and relative GS indices were strongly associated with MS, and the WHtR was the index most strongly associated with MS in the Korean population. In clinical practice, we recommend the use of anthropometric indices rather than GS indices to screen for MS based on the simplicity and cost-effectiveness of the measurement.

## Figures and Tables

**Table 1:** General characteristics of the participants in this study.

Variable	Men			Women		
	Healthy	MS	*p* Value ^a^	Healthy	MS	*p* Value ^b^
Participants (n)	5525	3742		7642	4006	
Age (years) ***	55.12 ± 0.17	57.11 ± 0.21	<0.001	53.92 ± 0.15	62.77 ± 0.22	<0.001
Anthropometric data						
Height (cm) ***	169.4 ± 0.11	169.8 ± 0.12	0.034	157.1 ± 0.08	154.9 ± 0.11	<0.001
Weight (kg) ***	67.35 ± 0.16	75.31 ± 0.20	<0.001	56.25 ± 0.11	62.85 ± 0.19	<0.001
WC (cm) ***	83.66 ± 0.12	92.16 ± 0.14	<0.001	77.25 ± 0.12	88.09 ± 0.17	<0.001
WHtR (ratio) **	0.49 ± 0.00	0.54 ± 0.00	<0.001	0.49 ± 0.00	0.57 ± 0.00	<0.001
BMI (kg/m2) ***	23.42 ± 0.04	26.08 ± 0.06	<0.001	22.77 ± 0.04	26.16 ± 0.06	<0.001
Systolic BP (mmHg) ***	118.6 ± 0.23	127.2 ± 0.32	<0.001	115.1 ± 0.21	129.0 ± 0.34	<0.001
Diastolic BP (mmHg) ***	77.22 ± 0.15	81.34 ± 0.23	<0.001	73.89 ± 0.13	76.98 ± 0.19	<0.001
Grip strength						
Absolute GS (kg) ***	38.06 ± 0.13	38.06 ± 0.15	0.980	22.72 ± 0.07	21.19 ± 0.11	<0.001
GS/weight (kg/Weight) ***	0.57 ± 0.00	0.51 ± 0.00	<0.001	0.41 ± 0.00	0.34 ± 0.00	<0.001
GS/BMI (kg/BMI) ***	1.64 ± 0.01	1.47 ± 0.01	<0.001	1.01 ± 0.00	0.82 ± 0.00	<0.001
GS/WHtR (kg/WHtR) ***	77.63 ± 0.29	70.63 ± 0.32	<0.001	46.75 ± 0.17	37.69 ± 0.21	<0.001
Geographic area **						
Urban	82.87 (1.21)	80.63 (1.35)	<0.001	85.09 (1.08)	79.52 (1.42)	<0.001
Rural	17.13 (1.21)	19.37 (1.35)		14.91 (1.08)	20.48 (1.42)	
Education level ***						
<= Elementary school	13.34 (0.54)	16.27 (0.69)	<0.001	17.94 (0.58)	47.05 (1.01)	<0.001
Middle school	11.55 (0.50)	13.98 (0.67)		11.54 (0.44)	14.82 (0.68)	
High school	33.4 (0.80)	33.84 (0.95)		38.62 (0.72)	26.35 (0.88)	
>= University	41.71 (0.97)	35.91 (1.08)		31.9 (0.82)	11.79 (0.64)	
Occupation type ***						
White-collar worker	17.56 (0.68)	15.72 (0.77)	<0.001	11.79 (0.45)	3.89 (0.35)	<0.001
Office worker	13.14 (0.60)	12.08 (0.63)		9.03 (0.37)	3.42 (0.35)	
Service worker	10.36 (0.54)	9.9 (0.61)		18.35 (0.55)	15.43 (0.74)	
Farmer or fisher	5.59 (0.47)	6.29 (0.54)		2.34 (0.24)	4.24 (0.48)	
Blue-collar worker	26.06 (0.80)	23.93 (0.87)		3.73 (0.28)	2.63 (0.31)	
Elementary occupations	8.44 (0.42)	7.65 (0.50)		10.85 (0.41)	13.67 (0.66)	
Unemployed (housewife, etc.)	18.85 (0.58)	24.42 (0.83)		43.91 (0.72)	56.73 (0.98)	
Household income level ***						
Low	13.08 (0.53)	17.13 (0.76)	<0.001	14.83 (0.53)	32.63 (0.91)	<0.001
Middle-low	23.25 (0.70)	24.38 (0.85)		22.72 (0.62)	27.58 (0.83)	
Middle-high	29.15 (0.74)	26.97 (0.91)		27.9 (0.64)	22.16 (0.78)	
High	34.52 (0.94)	31.52 (1.00)		34.56 (0.84)	17.62 (0.78)	
Smoking status ***						
Daily	33.57 (0.81)	36.55 (0.94)	<0.001	4.38 (0.29)	4.25 (0.39)	<0.001
Past	46.45 (0.79)	48.14 (0.97)		4.43 (0.27)	3.85 (0.33)	
Never	19.98 (0.63)	15.31 (0.68)		91.19 (0.4)	91.9 (0.50)	
Alcohol intake ***						
Yes	81.34 (0.64)	83.61 (0.75)	<0.001	66.06 (0.63)	52.43 (0.97)	<0.001
No	18.66 (0.64)	16.39 (0.75)		33.94 (0.63)	47.57 (0.97)	
Stress status **						
Extreme	2.84 (0.26)	3.92 (0.38)	<0.001	3.9 (0.26)	5.28 (0.41)	<0.001
High	18.43 (0.63)	18.73 (0.74)		19.82 (0.52)	18.89 (0.75)	
Slight	60.9 (0.78)	58.39 (0.93)		60.78 (0.63)	53.42 (0.90)	
Rare	17.82 (0.56)	18.96 (0.75)		15.5 (0.47)	22.41 (0.74)	
Physical activity status (days) ***						
0	65.79 (0.72)	71.48 (0.87)	<0.001	80.64 (0.54)	89.17 (0.57)	<0.001
1~2	10.24 (0.49)	8.54 (0.53)		7.4 (0.35)	3.62 (0.34)	
3~4	11.45 (0.51)	7.94 (0.51)		6.71 (0.35)	3.63 (0.34)	
>5	12.52 (0.50)	12.04 (0.62)		5.26 (0.29)	3.57 (0.32)	
Menopause status						
Yes				54.47 (0.73)	81.04 (0.80)	<0.001
No				45.53 (0.73)	18.96 (0.80)	
Blood profiles						
FPG (mg/dL) ***	100.0 ± 0.34	117.6 ± 0.63	<0.001	94.34 ± 0.18	114.7 ± 0.56	<0.001
HDL (mg/dL) ***	49.76 ± 0.17	42.69 ± 0.20	<0.001	57.27 ± 0.17	46.41 ± 0.19	<0.001
TG (mg/dL) ***	132.9 ± 1.77	236.4 ± 4.00	<0.001	98.24 ± 0.67	174.6 ± 2.09	<0.001
High BP ***						
No	75.87 (0.68)	28.41 (0.89)	<0.001	81.7 (0.50)	27.14 (0.83)	<0.001
Yes	24.13 (0.68)	71.59 (0.89)		18.3 (0.50)	72.86 (0.83)	
High FPG ***						
No	66.71 (0.76)	18.79 (0.79)	<0.001	80.88 (0.54)	25.32 (0.79)	<0.001
Yes	33.29 (0.76)	81.21 (0.79)		19.12 (0.54)	74.68 (0.79)	
Low HDL ***						
No	81.83 (0.61)	35.12 (0.96)	<0.001	68.06 (0.63)	12.88 (0.61)	<0.001
Yes	18.17 (0.61)	64.88 (0.96)		31.94 (0.63)	87.12 (0.61)	
High TG ***						
No	75.87 (0.68)	27.26 (0.85)	<0.001	89.99 (0.41)	45.31 (0.94)	<0.001
Yes	24.13 (0.68)	72.74 (0.85)		10.01 (0.41)	54.69 (0.94)	
High WC ***						
No	84.98 (0.55)	33.33 (0.92)	<0.001	86.85 (0.47)	31.33 (0.90)	<0.001
Yes	15.02 (0.55)	66.67 (0.92)		13.15 (0.47)	68.67 (0.90)	
Dominant hand						
Right	88.47 (0.54)	88.78 (0.60)	0.573	90.09 (0.41)	89.05 (0.56)	0.572
Left	4.96 (0.34)	5.13 (0.40)		4.32 (0.28)	4.9 (0.40)	
Both	6.57 (0.42)	6.09 (0.45)		5.58 (0.31)	6.05 (0.43)	

**: *p* < 0.01, ***: *p* < 0.001. These marks indicate *p* values for sex differences between all men and women. Continuous data are presented as the mean ± standard error (SE), and categorical data are presented as percentages (SEs). *p* values were obtained via Rao–Scott chi-square tests for categorical variables and a general linear model for continuous variables. Statistical differences between the healthy and MS groups are presented as *p* values ^a^ for men and *p* values ^b^ for women. Abbreviations. Healthy group: group without metabolic syndrome; MS group: metabolic syndrome group; GS: maximum handgrip strength of the dominant hand; BMI: body mass index; WC: waist circumference; WHtR: waist-to-height ratio; BP: blood pressure; FPG: fasting plasma glucose; HDL: high-density lipoprotein cholesterol; TG: triglyceride.

**Table 2:** Associations of anthropometric, absolute GS, and relative GS indices with MS in men.

Variable	Crude		Model 1		Model 2	
	OR	*p* Value	adj. OR	adj. *p* Value	adj. OR	adj. *p* Value
Anthropometry						
Height	1.05 (1.00–1.10)	0.035	1.17 (1.11–1.24)	<0.001	1.19 (1.13–1.26)	<0.001
Weight	2.41 (2.26–2.57)	<0.001	3.15 (2.92–3.40)	<0.001	3.36 (3.10–3.64)	<0.001
BMI	3.02 (2.80–3.26)	<0.001	3.33 (3.08–3.61)	<0.001	3.49 (3.21–3.79)	<0.001
WHtR	3.98 (3.65–4.35)	<0.001	3.99 (3.65–4.36)	<0.001	4.08 (3.72–4.46)	<0.001
Grip strength						
Absolute GS	1.00 (0.95–1.05)	0.98	1.13 (1.07–1.20)	<0.001	1.16 (1.09–1.23)	<0.001
GS/weight	0.53 (0.50–0.56)	<0.001	0.53 (0.50–0.57)	<0.001	0.52 (0.49–0.55)	<0.001
GS/BMI	0.57 (0.54–0.60)	<0.001	0.56 (0.53–0.60)	<0.001	0.55 (0.51–0.59)	<0.001
GS/WHtR	0.64 (0.60–0.67)	<0.001	0.62 (0.58–0.67)	<0.001	0.62 (0.58–0.66)	<0.001

The crude model was unadjusted. Model 1 was adjusted for age, and Model 2 was adjusted for age, geographic area, education level, occupation type, household income level, stress status, alcohol intake, smoking status, and physical activity status. Odds ratios (ORs) and *p* values were calculated via crude and adjusted analyses via complex sample binary logistic regression and were estimated with 95% confidence intervals.

**Table 3:** Associations of anthropometric, absolute GS, and relative GS indices with MS in women.

Variable	Crude		Model 1		Model 2	
	OR	*p* Value	adj. OR	adj. *p* Value	adj. OR	adj. *p* Value
Anthropometry						
Height	0.68 (0.65–0.71)	<0.001	1.02 (0.96–1.08)	0.536	1.10 (1.03–1.16)	0.002
Weight	2.24 (2.12–2.37)	<0.001	3.11 (2.91–3.33)	<0.001	3.08 (2.88–3.29)	<0.001
BMI	3.37 (3.15–3.60)	<0.001	3.48 (3.25–3.73)	<0.001	3.39 (3.17–3.63)	<0.001
WHtR	5.27 (4.87–5.71)	<0.001	4.49 (4.14–4.87)	<0.001	4.40 (4.05–4.79)	<0.001
Grip strength						
Absolute GS	0.73 (0.69–0.76)	<0.001	1.06 (1.01–1.13)	0.03	1.09 (1.03–1.16)	0.003
GS/weight	0.43 (0.40–0.45)	<0.001	0.52 (0.49–0.55)	<0.001	0.53 (0.50–0.56)	<0.001
GS/BMI	0.40 (0.38–0.42)	<0.001	0.51 (0.48–0.54)	<0.001	0.53 (0.50–0.56)	<0.001
GS/WHtR	0.41 (0.38–0.43)	<0.001	0.54 (0.51–0.58)	<0.001	0.57 (0.53–0.60)	<0.001

The crude model was unadjusted. Model 1 was adjusted for age, and Model 2 was adjusted for age, geographic area, education level, occupation type, household income level, stress status, alcohol intake, smoking status, physical activity status, and menopausal status. Odds ratios (ORs) and *p* values were calculated via crude and adjusted analyses via complex sample binary logistic regression and were estimated with 95% confidence intervals.

## Data Availability

The Korea National Health and Nutrition Examination Survey (KNHANES) data used in these analyses are available from the Korea Centers for Disease Control and Prevention (KCDC). Anyone can freely access the data https://knhanes.kdca.go.kr/knhanes/sub03/sub03_02_05.do (6 March 2024).
